# Ongoing outbreak of maternal parvovirus B19 infections in Germany since end of 2023: consequence of COVID‐19 pandemic?

**DOI:** 10.1002/uog.29197

**Published:** 2025-03-06

**Authors:** J. Jiménez Cruz, R. Axt‐Fliedner, C. Berg, F. Faschingbauer, K. O. Kagan, J. Knabl, A. Lauten, H. Lehmann, H. Stepan, M. Tavares de Sousa, S. Verlohren, U. Germer, J. Weichert, B. Strizek, A. Geipel

**Affiliations:** ^1^ Department of Obstetrics and Prenatal Medicine University Hospital of Bonn Bonn Germany; ^2^ Division of Prenatal Diagnosis and Therapy Justus‐Liebig‐Universität Gießen Gießen Germany; ^3^ Division of Prenatal Medicine and Gynecologic Sonography, Department of Obstetrics and Gynecology University of Cologne Cologne Germany; ^4^ Department of Gynecology and Obstetrics, Erlangen University Hospital Friedrich Alexander University of Erlangen‐Nuremberg Erlangen Germany; ^5^ Department of Gynecology and Obstetrics University of Tuebingen Tuebingen Germany; ^6^ Department of Obstetrics and Perinatal Medicine Hallerwiese Clinic Nuremberg Germany; ^7^ Erfurt Prenatal Diagnostic Centre Erfurt Germany; ^8^ Department of Obstetrics, Vivantes Clinic Neukoeln Berlin Germany; ^9^ Department of Obstetrics University Hospital of Leipzig Leipzig Germany; ^10^ Center for Prenatal Medicine Hamburg Hamburg Germany; ^11^ Department of Obstetrics, Charité‐Universitätsmedizin Berlin, Corporate Member of Freie Universität Berlin and Humboldt‐Universität Berlin Germany; ^12^ Department of Prenatal Medicine St Josef Hospital Regensburg Germany; ^13^ Department of Gynecology and Obstetrics, Division of Prenatal Medicine University Hospital of Schleswig‐Holstein Lübeck Germany

**Keywords:** COVID‐19 pandemic, fetal anemia, hydrops fetalis, infectious disease in pregnancy, intrauterine transfusion, parvovirus B19

## Abstract

**Objective:**

To investigate the ongoing parvovirus B19 (B19V) outbreak among pregnant women in Germany and its connection to the coronavirus disease 2019 (COVID‐19) pandemic.

**Methods:**

This retrospective cohort study analyzed anonymous data regarding serologically confirmed B19V infections during pregnancy between January 2014 and April 2024 across 13 major fetal medicine centers in Germany. We evaluated the yearly frequency of B19V cases, cases that underwent intrauterine transfusion (IUT), cases presenting with hydrops fetalis and cases of intrauterine fetal death (IUFD) related to B19V infection, and stratified these variables by event occurrence < 20 weeks' gestation or ≥ 20 weeks' gestation. Variables were compared across three subperiods: pre COVID‐19 pandemic, during the COVID‐19 pandemic and post COVID‐19 pandemic.

**Results:**

Data from 918 pregnant women with confirmed B19V infection revealed a significant B19V outbreak since the end of 2023. The mean ± SD number of annual cases was 57.3 ± 20*.7* pre COVID‐19, 20*.*3 ± 13*.*5 during COVID‐19 and surged to 384.8 ± 299*.*8 post COVID‐19 *(P* < 0*.*01). Correspondingly, the number of cases in which the fetus underwent IUT increased post COVID‐19. The proportion of B19V diagnoses made before 20 weeks' gestation increased from 32.3% pre COVID‐19 to 53.2% post COVID‐19 (*P* < 0.001).

**Conclusions:**

These results demonstrate an unforeseen increase in B19V infections during pregnancy after the COVID‐19 pandemic, with a consequent rise in B19V cases with fetal anemia. The introduced policies during the COVID‐19 pandemic reduced the B19V infection rate but likely conditioned the present ongoing upsurge. Counseling, early detection and access to specialized centers performing IUT are essential measures required to address this outbreak. © 2025 The Author(s). *Ultrasound in Obstetrics & Gynecology* published by John Wiley & Sons Ltd on behalf of International Society of Ultrasound in Obstetrics and Gynecology.

## INTRODUCTION

Parvovirus B19 (B19V) is a common viral infection that typically presents in childhood with mild non‐specific symptoms such as fever, headache or myalgia. Seven to 10 days after viremia, patients commonly develop a typical facial exanthema described as an erythema of the cheeks but sparing the central face, known as ‘slapped cheek’ appearance. After this eruption appears, it is presumed that the patient is no longer contagious. This self‐limiting disease typically heals without consequences in immunocompetent individuals. However, in susceptible pregnant women, B19V infection poses a substantial threat to fetal health. As B19V disrupts fetal erythropoiesis, it may lead to anemia, which can result in hydrops fetalis and severe heart failure. Furthermore, the virus can directly affect myocardial cells, causing inflammation in the fetal myocardium^1^. These findings (fetal anemia, hydrops fetalis and myocarditis) are characteristic of complicated fetal infection, leading to an increased risk of miscarriage and intrauterine fetal death (IUFD)^2^, ^3^, ^4^. Pregnancies affected by B19V require more rigorous fetal sonographic surveillance to detect the early signs of fetal anemia. When this condition is observed, intrauterine transfusion (IUT) can substantially reduce the risk of fetal death, although this intervention is not free of complications^5^, ^6^, ^7^.

Regarding its epidemiology, B19V infections occur according to a seasonal cycle, with annual epidemics of varying size occurring in the spring. Larger B19V epidemics have been shown to occur once every 4 years^8^.

The coronavirus disease 2019 (COVID‐19) pandemic significantly influenced the spread of various infectious diseases owing to changes in human behavior, healthcare access and public health measures^9^, ^10^, ^11^. Lockdowns, social distancing and the reallocation of healthcare resources have affected the transmission dynamics of numerous pathogens. In the case of B19V, many countries have reported a rebound of new infections identified among blood samples from blood banks in 2023 and 2024, after 3 years of a noticeable reduction in cases that coincided with the COVID‐19 pandemic period (2019–2022)^8^, ^12^, ^13^, ^14^, ^15^. Reports of increasing B19V infection rates in different regions of Europe since 2023^13^, ^16^ led to warning communication from the European Centre for Disease Prevention and Control^17^.

The aim of this study was to examine whether the characteristics of the current B19V outbreak differed from those of the previous infection waves. We studied the effect of the COVID‐19 pandemic on the yearly B19V case frequency and the outcomes of B19V infection in pregnant women in Germany between January 2014 and April 2024.

## METHODS

We retrospectively collected anonymous data regarding serologically confirmed maternal B19V infections between January 2014 and April 2024 from 13 major diagnostic and fetal medicine centers across all regions of Germany (Figure S1). Data were obtained from the respective center's digital database records. Data sampling was performed using the European Survey Platform EUSurvey^18^. From each center, data were collected regarding the yearly number of B19V cases, cases that underwent IUT due to relevant fetal anemia, cases presenting with hydrops and cases of IUFD related to B19V infection. No individual patient data were required. Each variable was stratified based on the gestational age (GA) at occurrence into < 20 weeks' gestation or ≥ 20 weeks' gestation. Only data from women with serologically confirmed B19V infection were included in the study, defined as documented IgM antibody seroconversion or a positive polymerase chain reaction test. For the analysis of the relationship of B19V with the COVID‐19 pandemic, three periods were defined: a pre‐COVID‐19 period, including cases between January 2014 and December 2019; a COVID‐19 period, including cases between January 2020 and December 2022 when lockdown and other hygienic measures (e.g. wearing masks in public) were implemented in Germany; and a post‐COVID‐19 period, including cases from January 2023 until April 2024. Since we evaluated annual case frequency and the first hygienic measures in Germany were implemented in March 2020, the entire year of 2020 was included in the COVID‐19 period as it was considered to be mostly under the influence of those measures.

### Statistical analysis

Results are expressed as mean ± SD for continuous variables and as *n* (%) or odds ratio (OR) with 95% CI for categorical variables. The Kruskal–Wallis test was used for the analysis of continuous variables to compare cases between each study period. Descriptive categorical variables were analyzed using the chi‐square test or Fisher's exact test, as appropriate. Statistical analysis was performed using SPSS statistical software version 27.0 (SPSS Inc., Chicago, IL, USA). *P* < 0.05 was considered statistically significant.

## RESULTS

**Figure 1:**
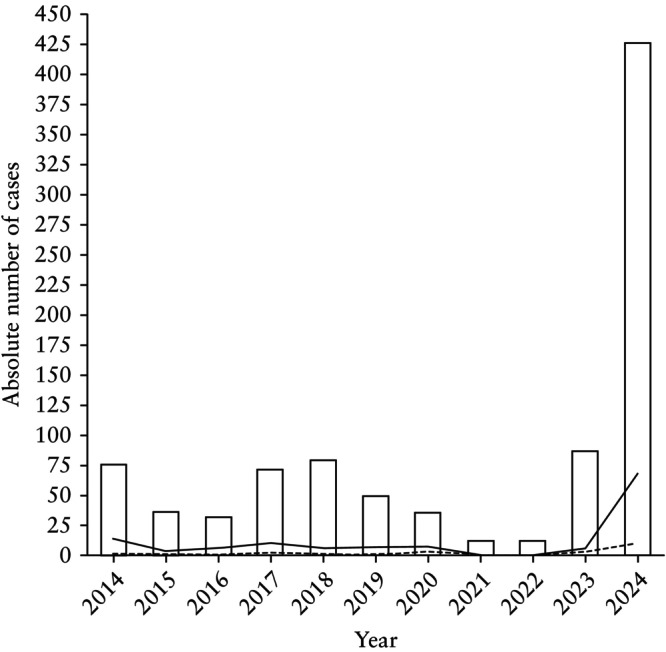
Cases of maternal parvovirus B19 (B19V) infection between January 2014 and April 2024, and cases in which intrauterine transfusion (IUT) was performed (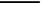) and those in which intrauterine fetal death (IUFD) occurred (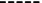).

A total of 918 pregnant women with confirmed maternal B19V infection were recorded during the study period. After a period of low annual case numbers during the COVID‐19 pandemic, there was a relevant increase in B19V infections (Figure 1). More cases of B19V were recorded in the first 4 months of 2024 than in the entire period between 2014 and 2022 (426 *vs* 405 cases). In addition, the number of cases undergoing IUT in January–April 2024 was higher than that in all previous years of the study period combined (68 *vs* 60 cases). Comparing the three defined subperiods, the mean ± SD number of cases per year was 57.3 ± 20.7 pre COVID‐19, 20.3 ± 13.5 during COVID‐19 and increased to 384.8 ± 299.8 cases per year post COVID‐19 (*P* < 0.01 for each pairwise comparison). The number of cases in which IUT was performed was in line with the number of B19V cases. While the annual mean ± SD number of fetuses undergoing IUT was 7.7 ± 3.5 pre COVID‐19, it declined to 2.3 ± 4.0 during COVID‐19 and reached 55.5 ± 43.9 post COVID‐19 (*P* < 0.01 for each pairwise comparison). Consequently, the annual rates of fetal hydrops and IUFD in each period also follow this trend. The average number of fetuses presenting with hydrops annually varied from 5.2 ± 2.1 pre COVID‐19 to 1.7 ± 2.9 during COVID‐19 and 42.0 ± 26.9 post COVID‐19 (*P =* 0.05), while the average number of annual IUFDs was 0.7 ± 0.8 pre COVID‐19, remained at 1 ± 1.7 during COVID‐19 and increased to 9.8 ± 7.4 post COVID‐19 (*P =* 0.11) (Table 1). It is noteworthy that the proportion of cases with IUT, IUFD or fetal hydrops did not change significantly over the years, except for in 2021 and 2022 when no B19V cases with IUT, fetal hydrops or IUFD were recorded (Table 1). Furthermore, no statistically significant difference was found in the cumulative frequencies of these variables between the three subperiods (Table 2).

**Table 1:** Cases of parvovirus B19 (B19V) infection in pregnant women over the study period, and annual rate of intrauterine transfusion (IUT), hydrops fetalis and intrauterine fetal death (IUFD) in pregnancies affected by B19V

Year	Cases (*n* = 918)	IUT	Hydrops	IUFD
2014	76	14 (18.4)	8 (10.5)	1 (1.3)
2015	36	4 (11.1)	3 (8.3)	0 (0)
2016	32	6 (18.8)	3 (9.4)	0 (0)
2017	71	9 (12.7)	6 (8.5)	2 (2.8)
2018	79	6 (7.6)	4 (5.1)	1 (1.3)
2019	50	7 (14.0)	7 (14.0)	0 (0)
2020	36	7 (19.4)	5 (13.9)	3 (8.3)
2021	12	0 (0)	0 (0)	0 (0)
2022	13	0 (0)	0 (0)	0 (0)
2023	87	6 (6.9)	9 (10.3)	3 (3.4)
2024*	426	68 (16.0)	47 (11.0)	10 (2.3)

Data are given as *n* or *n* (%). Percentages are calculated out of the number of cases in each year.

* Only cases from January to April 2024 are included.

**Table 2:** Maternal parvovirus B19 (B19V) infections, interventions and complications, stratified by gestational age at occurrence in three study subperiods

	Pre COVID‐19	During COVID‐19	Post COVID‐19	*P*
Maternal B19V infection	344	61	513	
< 20 GW	111/344 (32.3)	27/61 (44.3)	273/513 (53.2)	< 0.001
≥ 20 GW	233/344 (67.7)	34/61 (55.7)	240/513 (46.8)	< 0.001
IUT	46/344 (13.4)	7/61 (11.5)	74/513 (14.4)	0.780
< 20 GW	17/111 (15.3)	4/27 (14.8)	51/273 (18.7)	0.630
≥ 20 GW	29/233 (12.4)	3/34 (8.8)	23/240 (9.6)	0.637
Hydrops	31/344 (9.0)	5/61 (8.2)	56/513 (10.9)	0.586
< 20 GW	8/111 (7.2)	3/27 (11.1)	36/273 (13.2)	0.248
≥ 20 GW	23/233 (9.9)	2/34 (5.9)	20/240 (8.3)	0.687
IUFD	4/344 (1.2)	3/61 (4.9)	13/513 (2.5)	0.141
< 20 GW	2/111 (1.8)	3/27 (11.1)	13/273 (4.8)	0.094
≥ 20 GW	2/233 (0.9)	0/34 (0)	0/240 (0)	0.307

Data are given as *n* or *n*/*N* (%). Pre COVID‐19 was January 2014–December 2019, during COVID‐19 was January 2020–December 2022 and post COVID‐19 was January 2023–April 2024. GW, gestational weeks; IUFD, intrauterine fetal death; IUT, intrauterine transfusion.

**Figure 2:**
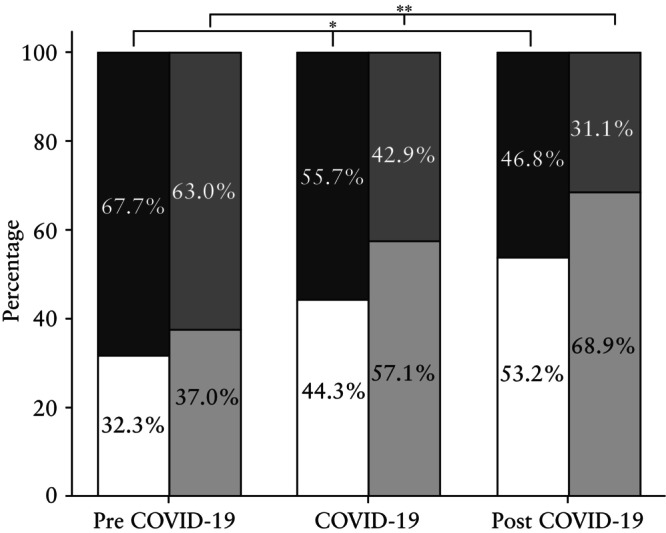
Distribution of cases of maternal parvovirus B19 infection diagnosed < 20 weeks' gestation (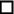) and ≥ 20 weeks' gestation (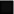) and proportion of cases that underwent intrauterine transfusion (IUT) < 20 weeks' gestation (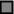) and ≥ 20 weeks' gestation (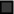), according to study subperiod. Pre COVID‐19 was January 2014–December 2019, during COVID‐19 was January 2020–December 2022 and post COVID‐19 was January 2023–April 2024. *P < 0.001; **P = 0.003.

When stratified by GA at the time of maternal B19V infection, statistically significant differences in the distribution of cases of B19V diagnosed < 20 weeks' gestation or ≥ 20 weeks' gestation were observed between the three subperiods. The proportion of cases diagnosed < 20 weeks' gestation increased constantly over time from 32.3% to 44.3% to 53.2% of cases pre COVID‐19, during COVID‐19 and post COVID‐19, respectively (*P <* 0.001). Consequently, a significant increase in the proportion of IUTs performed < 20 weeks' gestation, relative to the total number of IUTs, was observed from pre COVID‐19 to during COVID‐19 and post COVID‐19 (*P* = 0.003) (Figure 2). However, no statistical difference was observed between the three subperiods regarding the frequency of IUT, hydrops and IUFD when stratified by GA, in relation to the number of B19V cases in each GA range (Table 2).

With respect to fetal outcomes, diagnosis of B19V infection < 20 weeks' gestation and hydrops fetalis at the time of diagnosis resulted in a significant increase in the relative risk of IUT and IUFD. The ORs for IUT and IUFD < 20 weeks' gestation compared with ≥ 20 weeks' gestation were 1.75 (95% CI, 1.20–2.55) and 11.56 (95% CI, 2.6–50.14), respectively. The ORs for IUT and IUFD in cases with hydrops fetalis at the time of diagnosis compared to those without were 191.20 (95% CI, 86.99–420.23) and 62.18 (95% CI, 17.82–217.02), respectively.

## DISCUSSION

The present study shows an ongoing large increase in the incidence of B19V infections during pregnancy in Germany, requiring strict sonographic surveillance to detect potential signs of fetal compromise and consequently increasing the need for short‐term therapy. In the first 4 months of 2024, more cases of B19V infection were registered than in the entire period between 2014 and 2022 (426 *vs* 405 cases). Notably, if we extrapolate the data from these first 4 months to the entire year of 2024, the average number of B19V cases in 2024 would have been 677.5, which is about 13 times the pre‐COVID‐19 annual rate of B19V infection. This increase in cases follows significantly lower infection rates coinciding with the COVID‐19 pandemic. In addition, the absolute number of IUTs in 2024 was higher than that in all previous years of the study period combined (68 *vs* 60).

Nevertheless, in relation to the total number of cases, the relative proportions of IUT, hydrops fetalis and IUFD remained constant over time, suggesting stable rates of fetal transmission. Our data also show a constant increase in the proportion of B19V cases diagnosed before the 20^th^ week of gestation across the whole study period. This increase in diagnoses at an early GA might be due to the highlighted awareness during the ongoing B19V outbreak in the first quarter of 2024 or improved knowledge about the risks of B19V in pregnancy in general. It could be also speculated that, because of the reduction in circulating viruses during the COVID‐19 pandemic, fewer infants and mothers developed immunity after infection, leaving a larger proportion of the population susceptible.

Observation of the data over time helps to recognize that before the COVID‐19 pandemic smaller outbreaks of B19V occurred every 3–4 years. This cyclic augmentation of B19V incidence has been described previously and is well‐known to be normal for B19V^8^. Our data suggest that the present ongoing outbreak might have occurred after the expected peak in 2022 failed to materialize, likely due to the reduction in exposure to B19V because of the hygienic measures implemented to control the COVID‐19 pandemic, indirectly suggesting that these measures are also working to prevent B19V infections in pregnancy. It could be hypothesized that the reduction in infant immunization because of these measures led to an increase in B19V infections in subsequent years in this subgroup of the population, which might explain the increase in exposure of pregnant individuals to the virus. To support this hypothesis, it would be interesting to study if multiparous women are especially affected, as they are likely more often in contact with the virus through infection of previous children.

After a stable period with small cyclic rebounds between 2014 and 2019, no IUTs or IUFDs were registered in any of the included 13 centers in 2021 or 2022. This unexpectedly prolonged interval between peaks has also recently been described by a Danish group^12^. However, the presently observed incidence peak is extremely high and has never been observed before. This phenomenon has already been described in some countries by other groups^8^, ^12^, ^17^, ^19^, ^20^. Recent publications evaluating this outbreak describe an increase in cases in young children, adolescents and pregnant women^8^, ^15^, ^19^. The major issue in assessing the incidence and prevalence of B19V infection is that most countries do not perform systematic national surveillance^21^. Data are usually obtained from national blood banks, diagnostic laboratories or voluntary surveillance systems, as well as from local reports. For example, Russcher *et al*.^8^ reported a change in the epidemiology of B19V among the Dutch population using data obtained from blood banks, the voluntary sentinel surveillance network and hospital data from pregnant patients presenting with fetal hydrops or undergoing IUT. In a later correspondence^22^, they presented the first published data to show an increase in the number of IUTs, performed in four Northern European centers, and suggested that the proportion of fetal B19V cases with adverse outcomes is larger than expected based on existing literature and previous experiences. Although our data confirmed the increase in cases of severe fetal anemia in Germany, we have not reported an increase in IUT or adverse event rates. The proportions of IUT (14.4%), hydrops fetalis (10.9%) and IUFD (2.5%) in the post‐COVID‐19 period in our cohort were in line with the expected rates reported in the literature^4^. Thus, we are likely only witnessing the consequences of the high infection rates in young people and women of childbearing age, as suggested by Mor *et al*.^15^ after studying the virus causing the outbreak in Isreal and not finding any atypical strains^15^.

The data presented herein support previous observations that the necessity for IUT is more likely if B19V infection is diagnosed < 20 weeks' gestation^6^. As illustrated in Figure 2 and Table 2, a higher number of cases undergoing IUT in the post‐COVID‐19 period is most likely the result of early GA at infection because the proportion of IUTs in relation to the number of B19V cases in each GA range remained similar. This increase in diagnosis at an early GA could be related to early exposure or, more likely, be caused by improved awareness and knowledge regarding the risks of B19V in pregnancy, as suggested above. The distribution of cases stratified by GA at infection was only described in the cohort studied by Patalon *et al*.^19^, in which they also observed a significant increase in B19V diagnoses in the first trimester. The increase in early diagnoses could also explain the relatively high numbers of IUT observed in this cohort because early infection and the presence of hydrops fetalis have been described as risk factors for IUT and IUFD^4^, ^6^, ^23^.

Since there is no available vaccine or effective therapy for B19V, counseling, early detection and access to specialized centers for IUT are crucial in managing B19V infections during pregnancy because these measures can significantly reduce the risk of adverse fetal outcomes such as anemia, hydrops fetalis and death^24^, ^25^.

Some authors have suggested that IUT is related to abnormal neurosonographic findings in infants and fetuses who underwent IUT for B19V‐induced anemia^5^. Long‐term data, particularly those describing neurological development, are inconsistent. Some studies found that survivors of intrauterine B19V infection did not show relevant neurodevelopmental impairments^26^, ^27^, ^28^, but more recent studies have suggested slightly more neurological sequelae in these children^23^, ^29^. Considering that during the present ongoing outbreak the virus is affecting an exceptional number of fetuses, it would be useful to perform systematic follow‐up to explore the neurodevelopmental integrity of these infants in their first years of life to allow more accurate statements in the future.

This study has some limitations. Data acquisition was performed retrospectively and therefore selection bias cannot be ruled out. We tried to reduce this effect by not only including data from fetal therapy centers performing IUT, but also from diagnostic centers performing Doppler follow‐up after confirmed B19V infections and referring patients for IUT, if necessary. The number of cases with early hydrops fetalis due to B19V infection leading to miscarriage or IUFD is likely underrepresented in this cohort because these cases would not be seen in diagnostic centers. This effect is well known, and some authors estimate that B19V can be detected in more than 2% of early miscarriages or fetal deaths^30^.

In conclusion, an unusually high number of pregnant women have contracted B19V infection since the end of the COVID‐19 pandemic, leading to a large increase in fetal infections requiring targeted ultrasound monitoring and IUT. Despite higher infection rates, the proportion of complications such as fetal hydrops and IUFD remained stable within this German cohort. The relevant decrease in infections during the COVID‐19 pandemic period shows that the introduced public health policies were effective in reducing B19V infection at this time, but likely conditioned the present upsurge. Targeted follow‐up should be performed to explore the long‐term neurodevelopment of exposed infants because data regarding this issue are sparse.

## Supporting information


**Figure S1** Distribution of the participating centers across Germany.

## Data Availability

The data that support the findings of this study are available from the corresponding author upon reasonable request.
