# Supplementary figures and images for: Ongoing outbreak of maternal parvovirus B19 infections in Germany since end of 2023: consequence of COVID‐19 pandemic?
Source: Ultrasound Obstet Gynecol. 2025 Mar 6;65(4):456–61. doi: 10.1002/uog.29197 (PMC11961107; doi:10.1002/uog.29197)

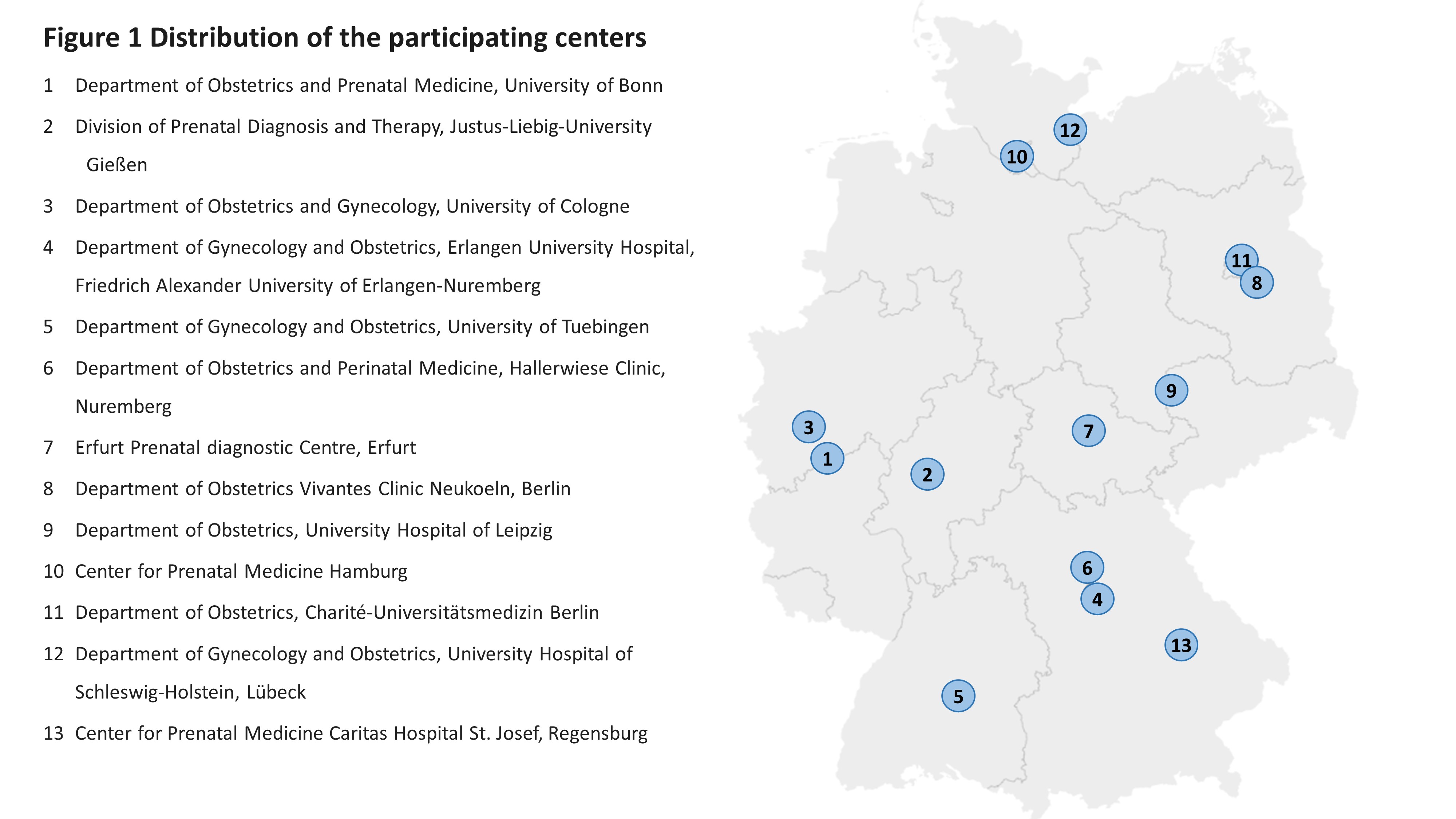

Supplement: Supplementary file 1 — Figure S1 Distribution of the participating centers across Germany. [file UOG-65-456-s001.jpg]
